# Comparative analyses of the biological characteristics, fluconazole resistance, and heat adaptation mechanisms of *Candida auris* and members of the *Candida haemulonii* complex

**DOI:** 10.1128/aem.02406-24

**Published:** 2025-03-26

**Authors:** Wei Xiao, Hao Zhou, Jian Huang, Caiyan Xin, Jinping Zhang, Huan Wen, Zhangyong Song

**Affiliations:** 1 Public Center of Experimental Technology, School of Basic Medical Sciences, Southwest Medical University 74647 , Luzhou, China; 2 Department of Medical Laboratory, Xuyong County People’s Hospital, Luzhou, China; 3 Hemodynamics and Medical Engineering Combination Key Laboratory of Luzhou, Luzhou, China; Chalmers tekniska hogskola AB, Gothenburg, Sweden

**Keywords:** *Candida auris*, *Candida haemulonii *complex, drug resistance, heat adaptation mechanism, pathogenicity

## Abstract

*Candida auris* and the emerging fungal pathogens comprising the *Candida haemulonii* complex (*C. haemulonii* and *C. duobushaemulonii*) are phylogenetically closely related. Notably, each can cause serious nosocomial infection and acquire multidrug resistance. We isolated various strains of *C. auris* and *C. haemulonii* complex from patient specimens. The results of growth curve analysis and the spot assay showed that *C. auris* was the most tolerant to high temperatures, while differences were found among the five strains of the *C. haemulonii* complex. We selected a representative strain from each of the three types of fungi that exhibit differences in heat resistance for further research. In addition, three strains were resistant to fluconazole, whereas sensitivity to common antifungal agents differed, as determined by the micro liquid-based dilution method. Moreover, *C. auris* was more virulent in *Galleria mellonella* than members of the *C. haemulonii* complex. In addition, there were notable differences in biofilm formation, hydrophobicity, adhesion, and enzyme activity among the three *Candida* strains. The results of transcriptomics, real-time quantitative PCR, and biochemical analyses showed that *C. auris* was most tolerant to heat stress due to the expression of genes in regulating pyruvate consumption and the accumulation of intracellular pyruvate. These results provide valuable references for further studies of the biological characteristics, pathogenesis, and treatment of *Candida* infection.

**IMPORTANCE:**

*Candida auris* and the *Candida haemulonii* complex are multidrug-resistant fungi that have emerged in recent years, posing a significant threat to human health. The biological characteristics of two strains of the *Candida haemulonii* complex and one strain of *C. auris* isolated from clinical patient samples were analyzed. Our primary focus was to compare the heat resistance between *C. auris* and the *C. haemulonii* complex, with a particular emphasis on understanding the differences in the heat resistance mechanisms. The main distinction between environmental and pathogenic fungi is that the latter can survive at human body temperature. Despite their close phylogenetic relationship, the *C. haemulonii* complex and *C. auris* exhibit significant differences in heat resistance. Studying these heat resistance mechanisms may aid in our understanding of the evolutionary process of environmental fungi transforming into pathogenic fungi.

## INTRODUCTION

Fungi are the main decomposers in natural ecosystems and have symbiotic relationships with a variety of organisms (1). A landmark paper estimated that there are 1.5 million species of fungi existing on Earth, of which less than 0.01% are human pathogens (2). Core body temperature is the main factor limiting human infection of pathogenic fungi (3). Fungal pathogens adapt to heat stress by regulation of a variety of signaling pathways and related transcription factors and secretion of fungal enzymes, including glutathione and trehalose, in addition to other heat-resistant protective substances, as well as adaptive changes to cell structures (4–6). Hence, elucidation of the heat-resistant mechanisms of pathogenic fungi would be helpful to prevent and control fungal infections (7). Recent studies have investigated the mechanisms of heat resistance in various pathogenic fungi, including *Aspergillus fumigatus*, *Candida albicans*, and *Cryptococcus neoformans* (8–11).

Nosocomial outbreaks of multidrug-resistant *C. auris* and members of the *C. haemulonii* complex (*C. haemulonii* and *C. duobushaemulonii*) have emerged as important risks for morbidity and mortality, particularly for hospitalized patients (12–14). *C. auris*, first isolated in Japan in 2008, is an emerging threat to public health globally, as five genetically distinct strains have simultaneously emerged on several continents (15). *C. auris* is more dangerous than other *Candida* species because of greater tolerance to environmental stressors, such as continued growth at 47°C, which is conducive not only to survival in harsh environments but also to overcome host defenses against colonization (16). A recent investigation speculated that human-induced global warming may have promoted the emergence of fungal pathogens (17). During the recent coronavirus disease pandemic, outbreaks of *C. auris* infection in intensive care units were reported in Colombia, Germany, and the United States (18, 19). The mortality rate of pathogenic *C. auris* reportedly ranges from 27% to 60% in hospitalized patients (20, 21). Notably, *C. auris* is closely related to the *C. haemulonii* complex and often misidentified (22, 23). Multidrug-resistant strains of *C. auris* and *C. haemulonii* complex are difficult to treat (24). *C. auris* mainly colonizes the skin and sometimes the intestinal, oral, and esophageal mucosae (25). In contrast, *C. haemulonii* complex mainly causes deep and superficial infections, such as catheter-related fungemia, peritonitis, and osteitis (26). Although the incidence of *C. auris* and *C. haemulonii* complex infections has continued to increase worldwide (27, 28), the mechanisms underlying pathogenicity, heat adaptation, and drug resistance remain unclear.

Therefore, the aim of the present study was to compare the heat tolerance, antifungal susceptibility, resistance to fluconazole (FLC), and virulence of *C. auris* and members of the *C. haemulonii* complex obtained from hospitalized patients. In addition, the similarities and differences of the heat resistance mechanisms of *C. auris* and *C. haemulonii* complex were further investigated to provide new insights for prevention and treatment.

## MATERIALS AND METHODS

### Strains and cultures

*C. haemulonii* var. *vulnera* (hereafter strain *ch*, isolated from blood samples), *C. duobushaemulonii* (hereafter strain *cd*, *cd2*, *cd3* isolated from blood samples and strain *cd1* isolated from skin specimens), and *C. auris* (hereafter strain *cau*, *cau03*, *cau13,* isolated from blood samples) were isolated from hospitalized patients (Table S1). Eight isolates were identified by sequencing of the nuclear ribosomal internal transcribed spacer region. The sequences were edited using Sequencher software (version 4.9; https://sequencher.software.informer.com/4.9/) and compared with sequences retrieved from the GenBank database (https://www.ncbi.nlm.nih.gov/genbank/) using the Basic Local Alignment Search Tool (https://blast.ncbi.nlm.nih.gov/Blast.cgi) (24). For subsequent experiments, all strains were activated on yeast extract-peptone-dextrose (YPD) medium (2% agar, 2% glucose, 2% peptone, and 1% yeast extract) for 48 h. Single colonies were selected and cultured in YPD liquid medium at 200 rpm for 18 h. Yeast cells were quantified using a Neubauer Counting chamber.

### Optimal incubation temperatures and quantification of colony-forming units (CFUs)

All activated strains were tested at concentrations of 10^3^, 10^4^, 10^5^, and 10^6^ cells/mL. Briefly, 1 mL aliquots were statically incubated in 2 mL Eppendorf tubes at 28°C, 37°C, 39°C, and 41°C within 0 h, 12 h, 24 h, 36 h, or 48 h. Meanwhile, 5 µL aliquots of the four fungal suspensions (10^3^, 10^4^, 10^5^, and 10^6^ cells/mL) were grown on YPD agar plates at 28°C, 37°C, 39°C, and 41°C, respectively. All samples after 48 h of incubation were imaged with a digital camera (Canon Inc., Tokyo, Japan) for comparisons of morphological characteristics. Based on differences in temperature tolerance among strains, an appropriate growth temperature for each strain was selected for subsequent experiments. Strain *cau* was incubated at 37°C, while strains *cd* and *ch* were incubated at 28°C. To ascertain the precise temporal point of a thermal response, the activated fungal suspensions were reintroduced into fresh YPD liquid medium at a concentration of 5 × 10^6^ cells/mL. For the establishment of static cultures, strain *cau* was incubated at 37°C, 39°C, and 41°C, whereas strains *cd* and *ch* were incubated at 28°C, 37°C, and 39°C. CFUs were quantified at each specified time point.

### Susceptibility to antifungal agents

Amphotericin B (AMB), anidulafungin (ANI), caspofungin (CAS), fluconazole (FLC), itraconazole (ITZ), isavuconazole (ISA), micafungin (MCF), posaconazole (PSZ), voriconazole (VOR), and 5-fluorocytosine (5-FC) were purchased from Shanghai Macklin Biochemical Co., Ltd. (Shanghai, China). The activities of the antifungal agents against all clinical and standard strains were evaluated using the broth microdilution method (Clinical and Laboratory Standards Institute [CLSI] M27-A4). *C. krusei* 6258 and *C. parapsilosis* 22019 (American Type Culture Collection, Manassas, VA, USA) were used as internal controls (Performance Standards for Antifungal Susceptibility Testing of Yeasts: CLSI supplement M60 2017; CLSI, Wayne, PA, USA). All organisms were cultured at least twice on antimicrobial-free YPD agar growth media and passaged to ensure purity and viability at 35°C. The concentration of the activated cells was adjusted to 2.5 × 10^3^ cells/mL. The wells of 96-well plates were loaded with 100 µL of the fungal sample with 100 µL of the diluted antifungal agent, and the plates were incubated at 35°C for 24 h. The minimum inhibitory concentration (MIC) for AMB was defined as the lowest concentration to prevent fungal growth. For the azoles, echinocandins, and flucytosine, the MIC was defined as the lowest concentration to decrease growth by 50%. The MIC was calculated by measuring absorbance at OD_600_ with a microplate reader.

### Adhesion, biofilm formation, and physiological activity assays

In accordance with a previously described method (29), the strains were incubated at various temperatures for 16 h, then centrifuged at 12,000 × *g* for 5 min, washed with phosphate-buffered saline (PBS), and resuspended in Roswell Park Memorial Institute (RPMI) 1640 medium at 1 × 10^6^ cells/mL. Aliquots of the fungal suspensions (200 µL) were added to the wells of 96-well plates and incubated at various temperatures for 4 h. Afterward, the supernatant was removed, and the wells were washed twice with PBS. Then, 100 µL of RPMI 1640 medium was added to each well and the plate was incubated for 24 h, followed by the addition of 2,3-bis(2-methoxy-4-nitro-5-sulfophenyl)−2H-tetrazolium-5-carboxanilide inner salt (XTT)-menadione solution and incubation for 2 h. Absorbance of the supernatant was measured at OD_490_.

The biofilm formation assay was performed in accordance with a previously described method (29). Briefly, 100 µL aliquots of fungal cell suspensions were added to the wells of 96-well plates. RPMI 1640 medium was used as a negative control. The plates were incubated at 28°C for 24 h. After incubation, the medium was carefully aspirated, and the wells were washed three times with PBS. To fix the biofilms, 100 µL of 10% formaldehyde solution was added to each well and the plates were incubated for 2 min at room temperature. Subsequently, 100 µL of 1 mg/mL crystal violet (CV) solution was added to each well and the plates were incubated for 30 min. The wells were then decolorized using 100 µL of 95% ethanol. Absorbance was measured at 630 nm using a microplate reader.

The biofilm mass was calculated using the following formula: biofilm biomass = OD sample – OD blank. OD sample is the absorbance (optical density) reading of the well containing fungal cells after crystal violet staining. OD blank is the absorbance of the blank control, which contains only the medium and staining reagents without any fungal cells. All experiments were performed in triplicate.

### Hydrophobicity test

In accordance with a previously described method (29), fungal cells (1 × 10^8^) were resuspended in PBS to a final volume of 2.25 mL and mixed with 0.75 mL of cyclohexane by vortexing for 3 min. After standing for 30 min, 200 µL of the aqueous phase was transferred to the wells of a 96-well plate. OD_600_ values were measured with a microplate reader. The hydrophobic value was calculated as (*A*_0_ *− A*_1_)/*A*_0_, where *A*_0_ and *A*_1_ are the initial and final absorbance at OD_600_, respectively.

### Aspartic-type protease and phospholipase activities

The bovine serum albumin (BSA) plate method is described in a previous report (30). Briefly, 5 µL aliquots of the fungal suspensions (5 × 10^6^ cells) were placed in the center of BSA plates (5% glucose, 0.025% KH_2_PO_4_, 0.0125% MgSO_4_, 5% agar, and 2.5% BSA) and incubated at the appropriate temperature for 120 h. The diameters of the colony ring and surrounding transparent ring were measured. The Pz value was calculated as the colony diameter/(colony diameter + clear ring diameter). The experiment was repeated three times for each strain, and the average Pz value was calculated. The phospholipase activity of the strain was determined using plates of 50% egg yolk solution and YPD solid medium (1:4). The other steps were consistent with the aspartic-type protease activity method (30).

### Virulence

Virulence of the fungal species to *Galleria mellonella* larvae was assessed in accordance with a previously described method (31). Briefly, the larvae were obtained from Laughing Monkey Biotech (China). Fungal cells were resuspended in PBS to a concentration of 5 × 10^6^ cells/mL. The abdominal cavity of each larva (body weight, 290–340 mg) was injected with 10 µL of the fungal cell suspension. A group of 10 larvae inoculated with the *cd* and *ch* strains was placed in a Petri dish at 28°C. Another group of 10 larvae inoculated with the *cau* strain was also placed in a Petri dish at 37°C to continue the infection procedure. After incubation for 24 h, each group was collected and homogenated in PBS (1:100 vol/vol), 10 µL aliquots were incubated at the appropriate temperature for 24 h, and the CFUs were counted.

Following infection, survival of the larvae was recorded for 6 days after injection with fungal suspension. The number of surviving larvae was recorded and photographed daily. Three replicate groups comprising 40 larvae each were assayed.

### Growth of fungal strains at various temperatures

The fungal solution was resuspended with fresh YPD liquid medium to 5 × 10^6^ cells /mL. Then, 1 mL of the resuspended fungal solution was added into 2 mL EP tube. Strain *cau* was incubated at 37°C, 39°C, and 41°C, while strains *cd* and *ch* were incubated at 28°C, 37°C, and 39°C, for a total duration of 120 h. Every 12 h, samples were collected by removing three tubes from each temperature condition. After fully mixing, 100 µL of each fungal suspension was transferred into triplicate wells of a 96-well plate. The growth of the fungal strains was then assessed using the XTT method, and growth curves were subsequently generated to visualize the data.

### RNA isolation and real-time quantitative polymerase chain reaction (RT-qPCR) analysis

To elucidate the mechanisms underlying resistance to heat stress, the expression levels of relevant genes were quantified by RT-qPCR analysis. In brief, fungal cells (1 × 10^7^) were grown in YPD liquid medium containing 16 µg/mL of FLC for 5 h. To clarify the thermal response mechanisms, strain *cau* was incubated at 39°C and 41°C for 12 h, strain *ch* at 37°C and 39°C for 1 h, and strain *cd* at 37°C and 39°C for 8 h. Total RNA was extracted with Yeast Processing Reagent (TaKaRa Biotechnology Co., Ltd., Dalian, China) and then reverse transcribed into complementary DNA using TB Green Premix Ex Taq II polymerase (TaKaRa Biotechnology Co., Ltd.). Primers for RT-PCR were designed using the National Center for Biotechnology Information website (https://www.ncbi.nlm.nih.gov/). The primers are listed in Table S2. The *β-actin* gene (*ACT1*) was used as an internal standard.

### Transcriptome sequencing and analysis

The specific heat resistance mechanism of the three strains at each time point was investigated. Strain *cau* was incubated at 39°C and 41°C for 12 h, strain *ch* at 37°C and 39°C for 1 h, and strain *cd* at 37°C and 39°C for 8 h. After centrifugation, the strains were precipitated and frozen in liquid nitrogen. Transcriptome sequencing was conducted by Qingdao Bimaike Biological Co., Ltd. (Qingdao, China). After qualification, the library was sequenced using the NovaSeq 6000 platform (Illumina, Inc., San Diego, CA, USA). Data analysis was performed using the BMK Cloud Bioinformatics Analysis Platform (https://www.bmkgene.com/bmkcloud-bioinformatics-analysis-platform/). Briefly, clean data were obtained by filtering incorrect, corrupted, incorrectly formatted, duplicate, and incomplete data within the data set. The obtained sequences were aligned against a reference genome (*Candida_auris*, GCA_003013715.2; *Candida_haemulonis*, GCA_002926085.1; *Candida_duobushaemulonii*, GCA_002926055) (16, 32). After the mapped data were obtained, library quality assessment, structural level analysis, differential expression analysis, gene functional annotation, and functional enrichment were performed. The detailed methods and gene functional annotation are described in a previous report by our group (33). Raw sequence data were deposited in the Beijing Institute of Genomics Genome Sequence Archive (no. CRA011199). Genes with fold change ≥2 and false discovery rate <0.01 were considered significantly differentially expressed.

### Measurements of intracellular reactive oxygen species (ROS) and pyruvate

Strain *cau* (5 × 10^6^ cells/mL) was cultured at 37°C, 39°C, and 41°C; strain *ch* was cultured at 28°C, 37°C, and 39°C; and strain *cd* was cultured at 28°C, 37°C, and 39°C, respectively. At each time point, the cells were collected by centrifugation at 12,000 × *g* for 2 min, washed twice with PBS, and then resuspended to 5 × 10^7^ cells/mL. Following the addition of a probe (2′,7′-dichlorodihydrofluorescein diacetate at 40 µmol/L), incubation was continued for 30 min. The fluorescence intensity, reflecting the intracellular ROS level, was detected at an excitation wavelength of 488 nm and an emission wavelength of 525 nm (29).

Meanwhile, at each time point, the cells were collected by centrifugation at 12,000 × *g* for 2 min and washed twice with PBS. Then, the supernatant was discarded and the cell pellet was ground in liquid nitrogen. The pyruvate content was measured using a commercial kit (Beijing BoxBio Biotechnology Co., Ltd. Beijing, China). Pyruvate was prepared at three concentrations: high (YPD liquid medium supplemented with 6 mM pyruvate), normal (normal YPD liquid medium), and low (YPD liquid medium supplemented with 4 µg/mL of the pyruvate kinase inhibitor PKM2-IN-1; MedChemExpress, Monmouth Junction, NJ, USA). Changes to ROS and pyruvate levels at different time points of heat stress were measured as described in a previous study (34). In addition, the CFUs were quantified and the survival rate was calculated.

### Statistical analysis

Statistical analysis was performed using GraphPad Prism 9 software (GraphPad Software, Inc., San Diego, CA, USA). Survival of the *G. mellonella* larvae was assessed with the Mantel-Cox test. The *t*-test and one-way analysis of variance were used to determine the significance of differences between two groups and among three or more groups, respectively. A probability (*P*) value <0.05 was considered statistically significant.

## RESULTS

### Strain identification and optimal growth temperature

**Fig 1:**
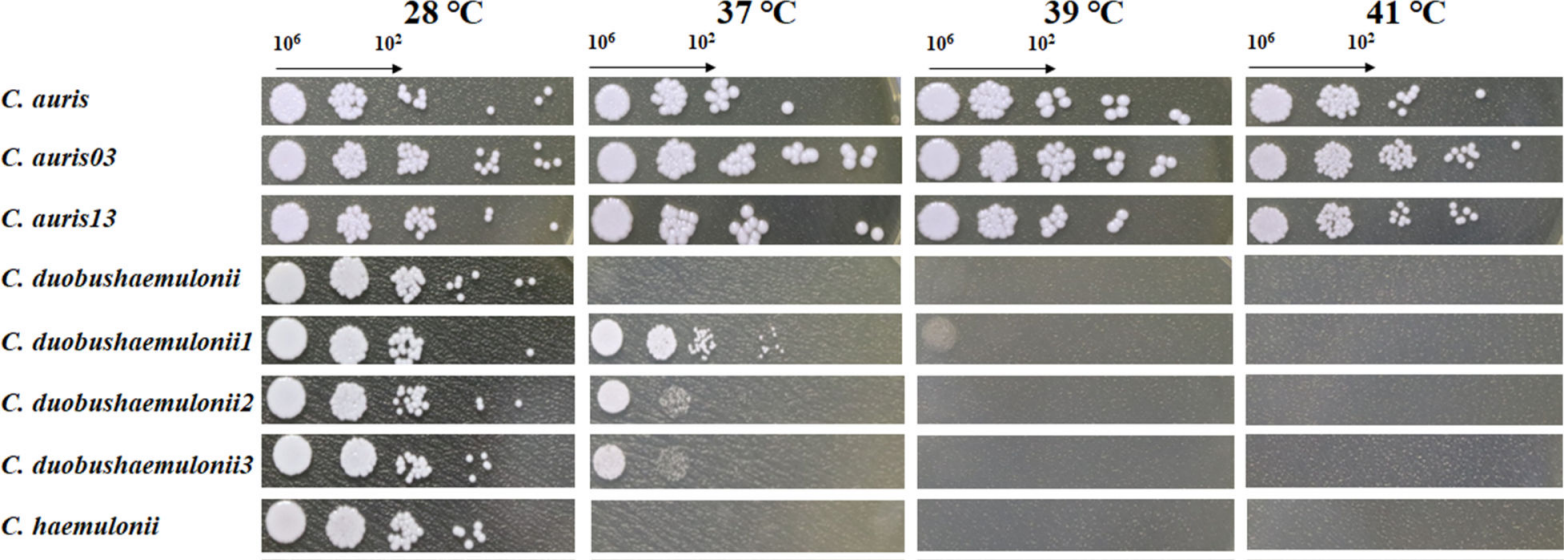
Tolerance to various temperatures of *C. auris* and members of the *C. haemulonii* complex.

We obtained multiple strains of *C. auris* and *C. haemulonii* complex from clinical samples and first analyzed their heat resistance. The yeast spotting assay was performed to determine the appropriate growth temperature of the four isolated strains. As shown in Fig. 1, *C. auris* strains can withstand temperatures up to 41°C; however, the colonies diminish in size as the temperature incrementally increases. The optimal culture temperature of the *C. haemulonii* complex is reportedly 37°C (35, 36). In this study, however, five *C. haemulonii* complex strains were able to grow at 28°C. As the temperature increases to 37°C, the *cd* and *ch* strains exhibit inhibited growth. At 39°C, only the high-concentration *cd1* strain can grow. At 41°C, no *C. haemulonii* complex strains grow. Overall, *C. auris* strains were the most resistant to heat, followed by strains *cd1*, *cd2,* and *cd3*, while strains *cd* and *ch* were the least resistant. We randomly selected three strains (*cau*, *cd*, and *ch*) from *C. auris* and *C. haemulonii* complex, which exhibit significant differences in heat resistance, for further research in this study. Other strains’ data will be presented in other articles.

### Drug susceptibility of tested strains

At present, the sensitivity of *C. auris* and *C. haemulonii* complex to antifungal agents remains unclear (13). The drug susceptibility results of the three strains were confirmed by reference to the Centers for Disease Control and Prevention and other reports in the literature (37, 38). The results showed that strains *cd* and *ch* exhibited reduced susceptibility to FLC, VRZ, ITZ, PSZ, and ANI (Table 1), while strain *cau* exhibited reduced susceptibility to FLC, VRZ, ITZ, PSZ, CAS, MCF, and ANI.

**TABLE 1:** Sensitivity of *C. auris* and *C. haemulonii* complex to common antifungal agents

Strain	MIC (μg/mL) of:									
	FLZ	VRZ	ITZ	PSZ	ISA	AMB	5-FC	CAS	MCF	ANI
*cau*	>256	32	>64	>32	0.5	0.5	0.5	>16	>32	>16
*cd*	>256	128	>64	>32	>64	0.5	0.5	0.25	0.0625	4
*ch*	>256	128	>64	>32	16	0.5	>64	>16	0.0625	>16

### Differences in pathogenicity and expression patterns of virulence factors

**Fig 2:**
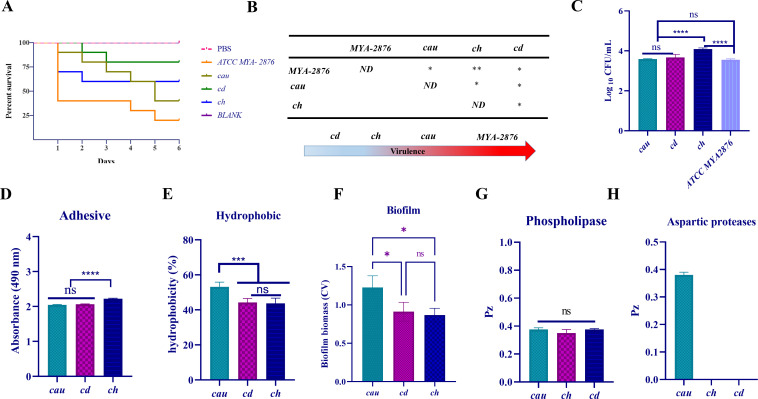
Virulence and pathogenicity of three strains. (**A**) Survival curve of *G. mellonella* larvae. (**B**) Analysis of the significant difference of survival and comparison of virulence. (**C**) Statistical differences were observed in the fungal load in a model of *G. mellonella* infection among the four isolates. (**D**) Comparison of surface adhesion among the three strains. (**E**) Comparison of surface hydrophobicity among the three strains. (**F**) Comparison of biofilm formation among the three strains. (**G**) Phospholipase activity. Hydrolytic enzyme activity was determined by calculating the Pz value. (**H**) Comparison of aspartase activity among the three strains. (*P < 0.05; **P < 0.01; ***P < 0.001; ****P < 0.0001; ND, not determined; ns, not significant).

The virulence of the three strains was analyzed with an infection model of *Galleria mellonella* larvae with *C. albicans* strain MYA-2876 as a positive control. Analysis of survival curves and fungal loads identified differences in virulence among the tested strains (Fig. 2A and B). The results showed that *C. albicans* strain MYA-2876 was the most virulent, followed by strains *cau* and *ch*, while strain *cd* was the least virulent. There were also differences in the fungal loads in *G. mellonella* larvae among the tested strains (Fig. 2C). The results showed that strains *cd* and *ch* produced the highest fungal loads, although there was no statistical difference, followed by strains *cau* and finally *C. albicans* strain MYA-2876, while there was no statistical difference among the latter three groups. Although strains *cd* and *ch* produced the highest fungal loads, strains *cau* and MYA-2876 had the highest mortality rates.

**Fig 3:**
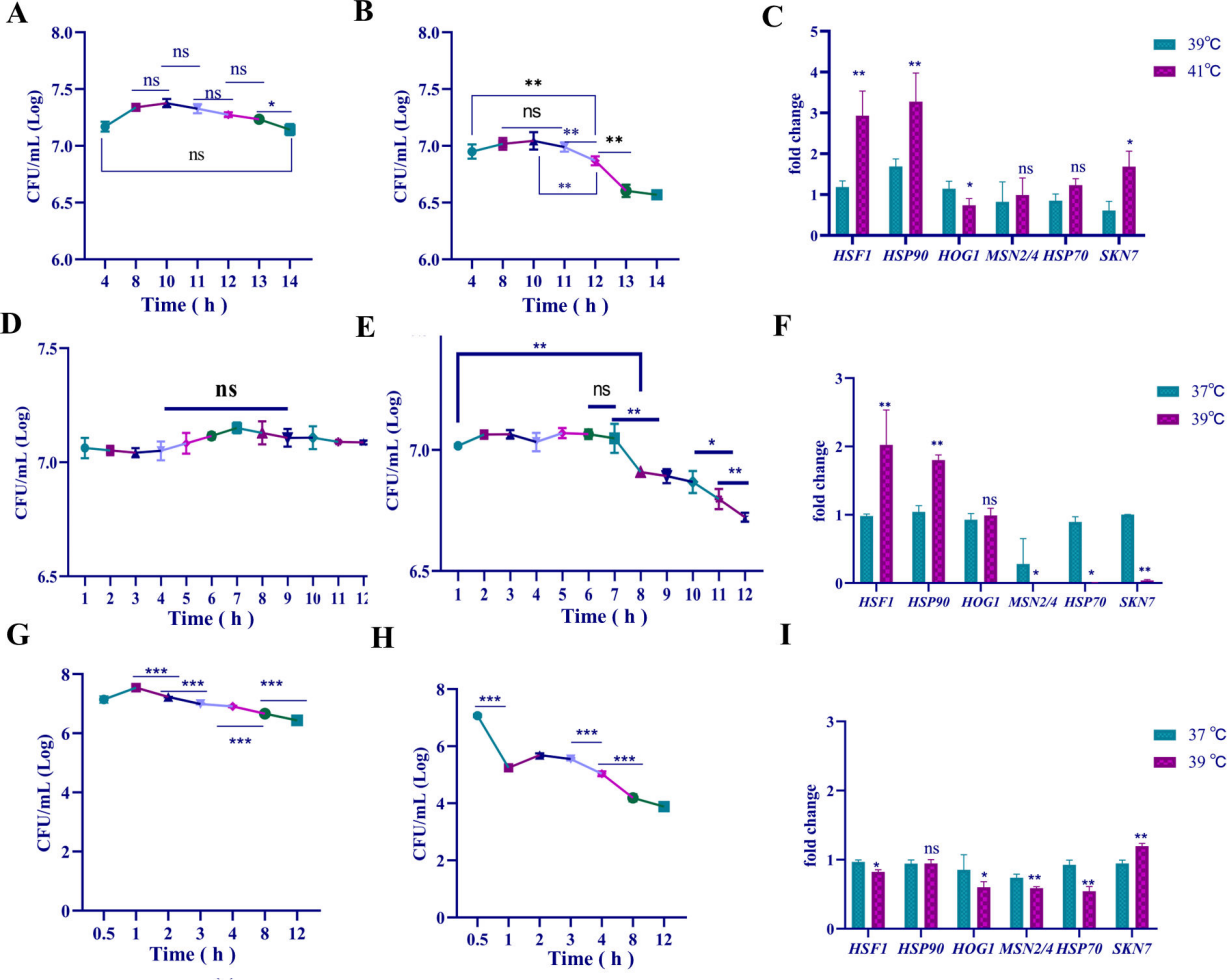
Changes to CFU count and expression levels of common genes related to heat tolerance. Population of strain *cau* cells in response to temperatures of (**A**) 39°C and (**B**) 41°C. (**C**) Fold change of genes related to heat tolerance in strain *cau*. Population of strain *cd* cells in response to temperatures of (**D**) 37°C and (**E**) 39℃. (**F**) Fold change in expression levels of genes related to heat tolerance in strain *cd*. Population of strain *ch* cells in response to temperatures of (**G**) 37°C and (**H**) 39°C. (**I**) Fold change in expression levels of genes related to heat tolerance in strain *ch*. Data are the mean  ±  standard deviation of three independent experiments. (*P  <  0.05; **P  <  0.01; ***P  <  0.001; ns, not significant).

To further confirm the main virulence factors of the three strains, the expression levels of virulence factors, including adhesion, hydrophobicity, biofilm-forming ability, and secretion of phospholipase and aspartase, were compared. The results showed that strain *ch* had the strongest adhesion ability, while there was no significant difference among strains *cau* and *cd* (Fig. 2D). Cell surface hydrophobicity is a non-specific virulence factor of pathogenic fungi and is classified at three different levels (39). Strains *cau*, *cd*, and *ch* were moderately hydrophobic. Strain *cau* had stronger hydrophobicity than strains *cd* and *ch* (Fig. 2E).

Strain *cau* strain exhibited the strongest biofilm formation ability, while there were no significant differences among strains *cd* and *ch* (Fig. 2F). In addition, *Candida* sp. also secrete molecules into the extracellular environment that directly/indirectly influence virulence (40). Therefore, the phospholipase and aspartase activities of the test strains were compared by calculating the Pz values, where a higher Pz value indicates lower secretory enzyme activity (41). The results showed that there was no significant difference among strains *cau*, *cd*, and *ch* (Fig. 2G). Moreover, only strain *cau* secreted aspartase, although there was no significant difference in Pz values (Fig. 2H).

### Differences in growth activity, thermal response time, and thermal response mechanism

Strains *cau*, *cd*, and *ch* were collected from the same types of samples to assess survival at core body temperature (37°C) and fever body temperature (39°C and 41°C). First, the XTT method was used to measure the physiological activity of the strains in response to heat stress for 120 h. As shown in Fig. S1, after 12 h of incubation, proliferation of strains *cau*, *cd*, and *ch* stabilized and then gradually stalled. The growth curves of strain *cau* cultured at 39°C and 37°C were similar with no significant decrease in growth at 41°C. However, proliferation of strains *cd* and *ch* was inhibited at 37°C and 39°C. Meanwhile, proliferation of strains *cd* and *ch* had slightly stagnated at a high concentration (Fig. S1B and C). In addition, during the 12 h preceding incubation at core body and fever body temperatures, strains *cau*, *cd*, and *ch* exhibited significant damage to the cell membrane with a decrease in hydrophobicity (data not shown). In short, the rapid decrease in proliferation at higher temperatures indicates that the strains reached a critical point of tolerance.

To confirm the exact point of the thermal response, CFUs were quantified at different time points of incubation at 37°C, 39°C, and 41°C within 12 h. The rapid decline in the population of the strain at higher stress temperatures indicated that the tolerance threshold was reached, leading to a collapse of the self-regulation system and gradual apoptosis. Maintenance of normal population fluctuation suggested that the strain can still tolerate lower stress temperatures. The time points to study the heat resistance mechanisms were by observing changes to the population size of the strains.

The expression levels of common heat-resistant genes of three strains were analyzed at different time points. The results showed that proliferation of strain *cau* did not significantly decrease at 39°C for 12 h, but it sharply decreased at 41°C (Fig. 3A and B). As compared to 39°C for 12 h, the expression levels of heat shock factor 1 (*HSF1*), heat shock protein 90 (*HSP90*), and kinase-regulated stress-responsive transcription factor 7 (*SKN7*) were significantly up-regulated, while expression of mitogen-activated protein kinase HOG1 (*HOG1*) was down-regulated at 41°C for 12 h (Fig. 3C). There were notable differences in the proliferation of strain *cd* at 37°C vs. 39°C for 8 h (Fig. 3D and E). As compared to incubation at 37°C for 8 h, the expression levels of *HSF1* and *HSP90* were up-regulated in strain *cd*, while the expression levels of *MSN2/4* (C_2_H_2_-like zinc-finger transcriptional factor), *HSP70*, and *SKN7* were down-regulated at 39°C for 8 h (Fig. 3F). There were also notable differences in gene expression levels of strain *ch* at 37°C vs. 39°C for 1 h (Fig. 3G and H). As compared to incubation at 37°C for 1 h, *SKN7* expression was up-regulated in strain *ch*, while expression of other genes related to heat resistance, with the exception of *HSP90*, were down-regulated after incubation at 39°C for 1 h (Fig. 3I). Together, these results indicate differences in responses to heat stress among the three strains. Therefore, further analysis of the specific heat resistance mechanism of the three strains at each time point was conducted.

### Heat response mechanism of strain *cau*

**Fig 4:**
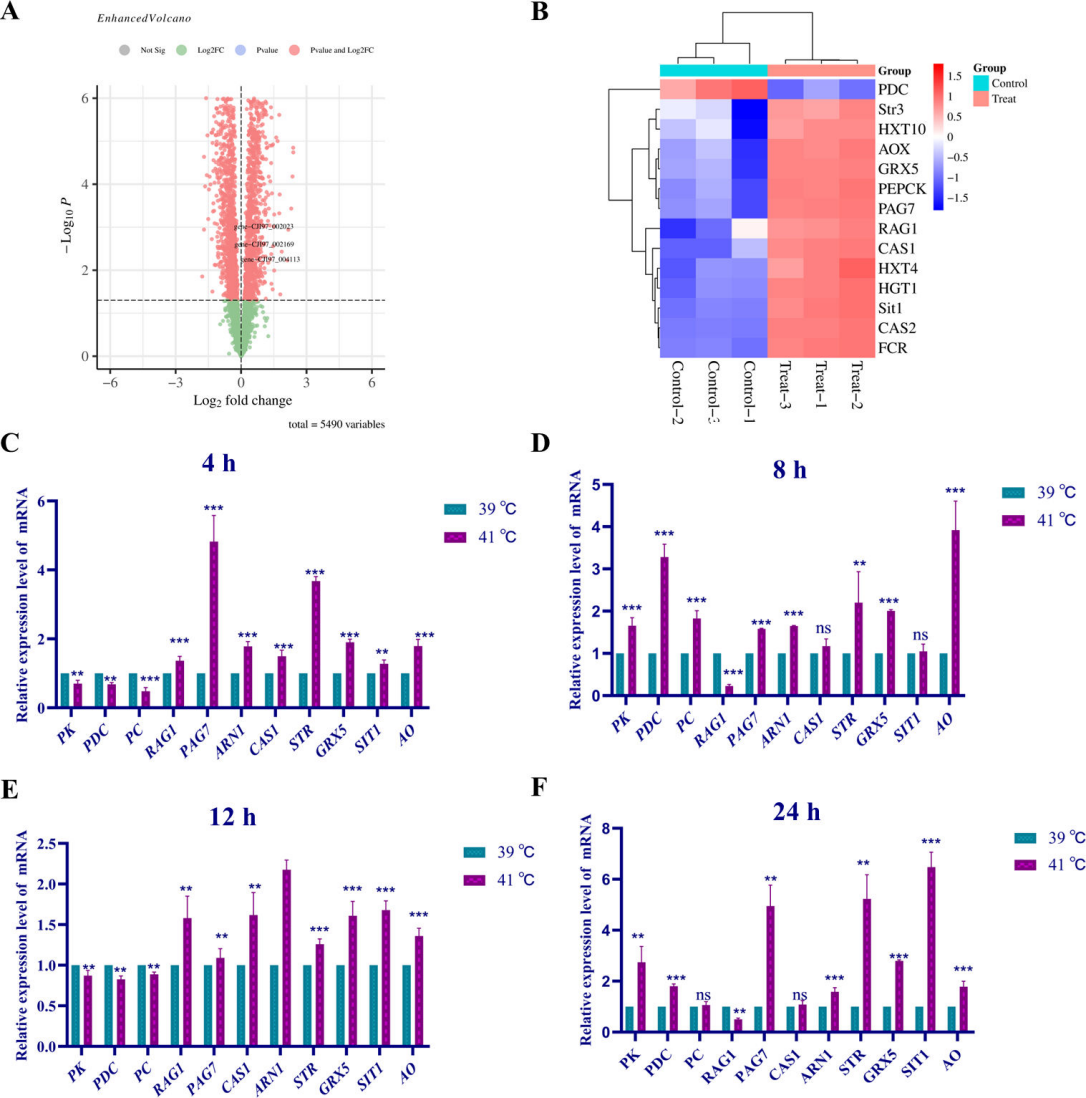
Transcriptome analysis and differential gene expression of strain *cau* in response to heat stress. (**A**) Volcano map of the differentially expressed genes of strain *cau* cultured at 39°C vs. 41°C for 12 h. (**B**) Heat map of differentially expressed genes of strain *cau*. The variation of heat-resistant functional differential gene in the *cau* strain after incubation at appropriate stress temperature for (**C**) 4 h, (**D**) 8 h, (**E**) 12 h, and (**F**) 24 h, respectively. Data are the mean  ±  standard deviation of three independent experiments. (**P  <  0.01; ***P  <  0.001; ns, not significant).

Transcriptome analysis was performed to identify genes regulating the heat response of strain *cau*. In total, 627 differentially expressed genes were identified for strain *cau* cultured at 39°C vs. 41°C for 12 h (Fig. 4A). The differentially expressed genes were concentrated in the glucose metabolism and iron transporter pathways (Fig. 4B). Further analysis found that genes associated with high-affinity glucose transporters (*HGT1* [CJI97_005618]), low-affinity glucose transporters (*RAG1* [CJI97_002023], *HXT10* [CJI97_002169], and *HXT4* [CJI97_001782]), and phosphoenolpyruvate carboxykinase (CJI97_002722) were significantly up-regulated, while expression of the pyruvate decarboxylase gene (CJI97_001552) was significantly down-regulated. Moreover, the expression levels of genes related to the iron absorption pathway (*PAG7* [CJI97_004534], *CAS1* [CJI97_002133], and *CAS2* [CJI97_004535]), transmembrane proteins (*Str3* [CJI97_004113], *Sit1* [CJI97_002301], and *ARN1* [CJI97_001760]), iron reductase (CJI97_004532), mitochondrial glutathione (*GRX5* [CJI97_005515]), and mitochondrial alternative oxidase (CJI97_002591) were up-regulated. Moreover, the results of RT-qPCR analysis of the gene expression levels of strain *cau* at various time periods in response to heat stress were consistent with the transcriptome data (Fig. 4C through F).

### Heat response mechanism of strain *cd*

**Fig 5:**
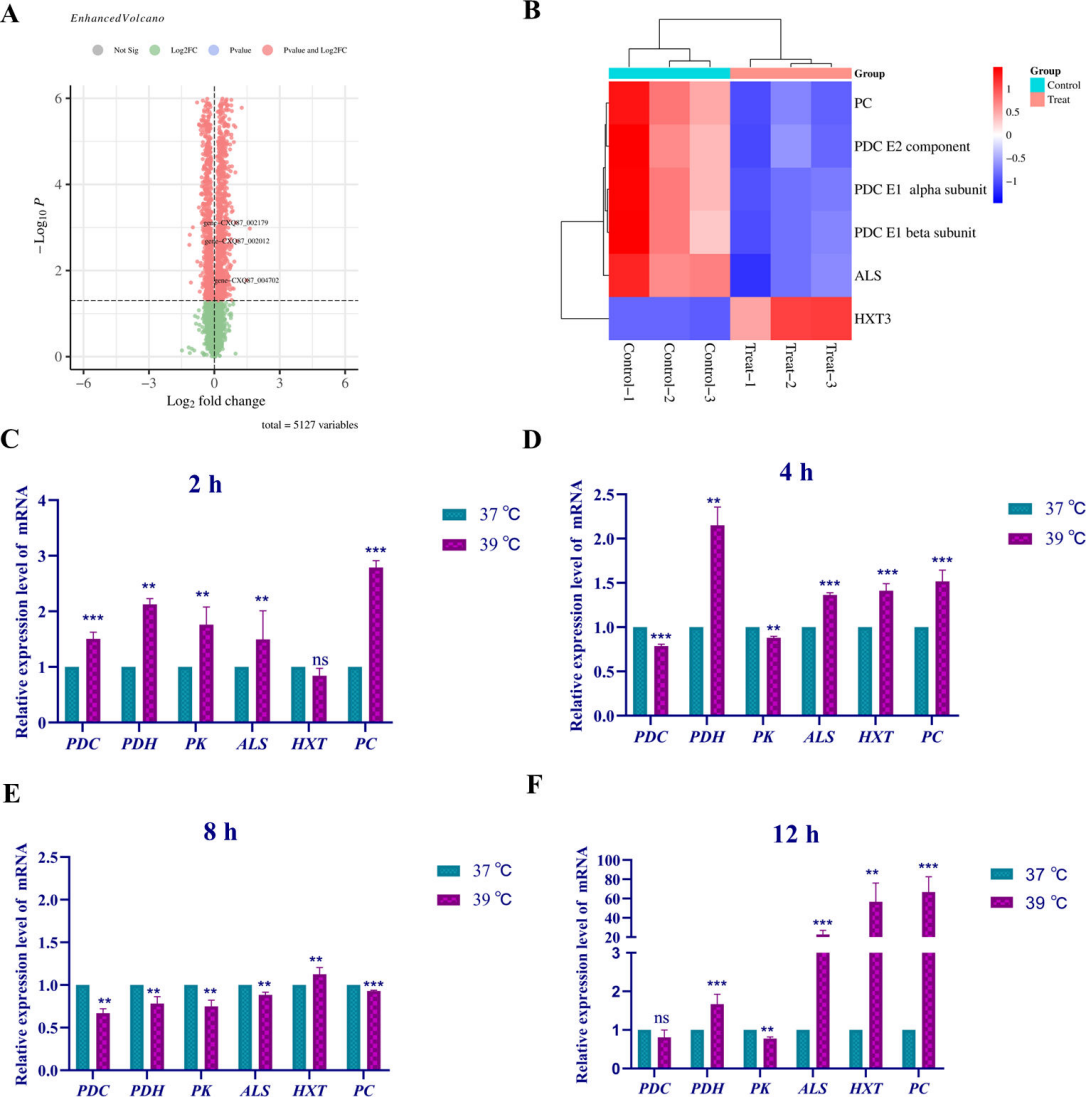
Transcriptome analysis and differential gene expression of strain *cd* in response to heat stress. (**A**) Volcano map of the differentially expressed genes of strain *cd* cultured at 37°C vs. 39°C for 8 h. (**B**) Heat map of differentially expressed genes of strain *cd*. The variation of heat-resistant functional differential gene in the *cd* strain after incubation at appropriate stress temperature for (**C**) 2 h, (**D**) 4 h, (**E**) 8 h, and (**F**) 12 h, respectively. Data are the mean  ±  standard deviation of three independent experiments. (**P  <  0.01; ***P  <  0.001; ns, not significant).

In total, 473 differentially expressed genes were identified for strain *cd* cultured at 37°C vs. 39°C for 8 h (Fig. 5A). Differentially expressed genes associated with the pyruvate dehydrogenase complex (*PDHA1* [CXQ87_004390], *PDHB1* [CXQ87_003966], and *PDHX* [CXQ87_000827]), *ALS* (CXQ87_000449), and *PC* (CXQ87_003960) were down-regulated, while the expression of the low-affinity glucose transporter gene *HXT3* (CXQ87_002560) was up-regulated (Fig. 5B). The results of RT-qPCR analysis of the gene expression levels of strain *cd* at various time periods in response to heat stress were consistent with the transcriptome data (Fig. 5C through F).

### Heat response mechanism of strain *ch*

**Fig 6:**
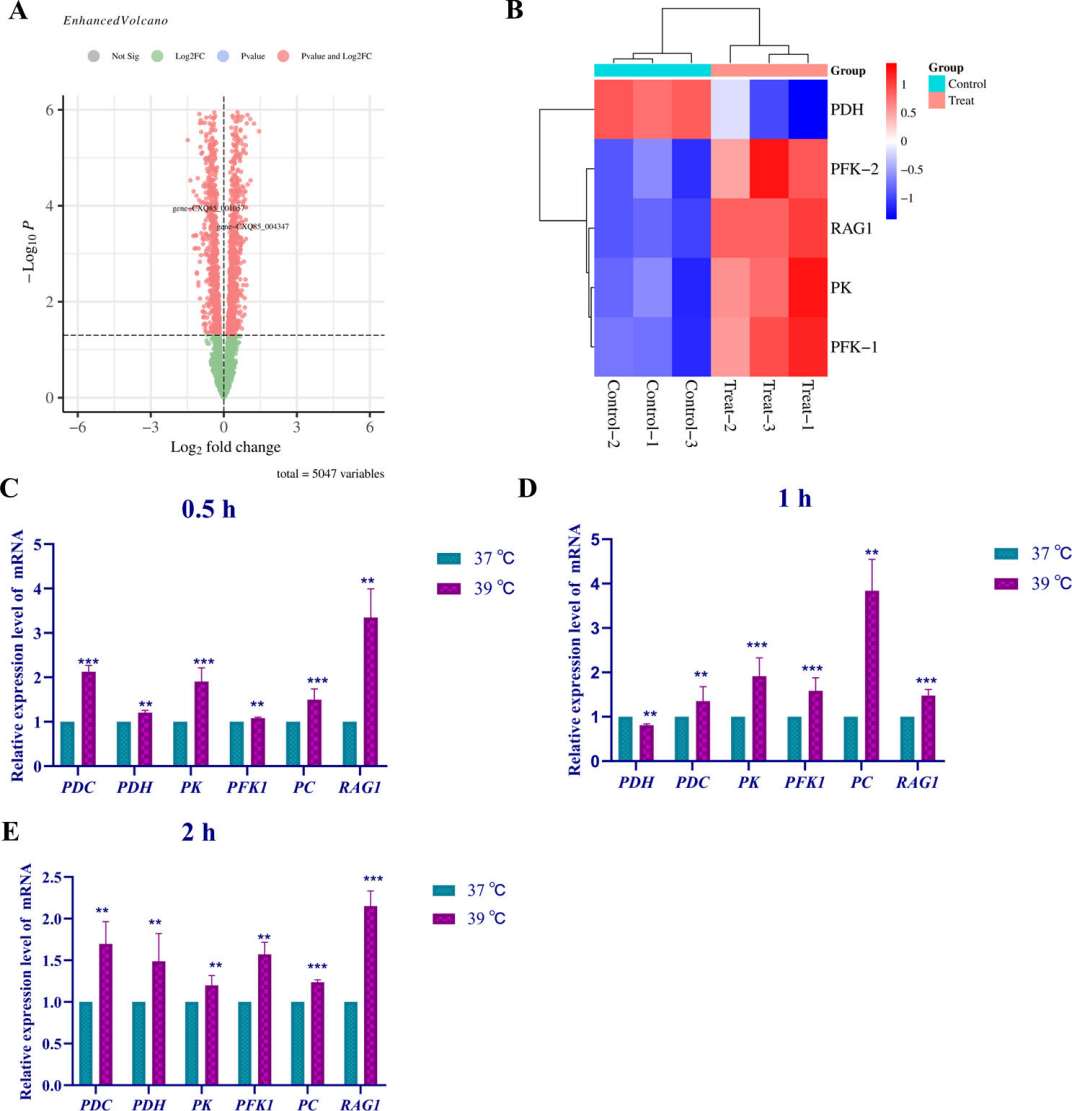
Transcriptome analysis and differential gene expression of strain *ch* in response to heat stress. (**A**) Volcano map of differentially expressed genes of strain *ch* cultured at 37°C vs. 39°C for 1 h. (**B**) Heat map of differentially expressed genes of strain *ch*. Variation in differentially expressed genes of strain *ch* at (**C**) 0.5 h, (**D**) 1 h, and (**E**) 2 h, respectively. Data are the mean  ±  standard deviation of three independent experiments. (**P  <  0.01; ***P  <  0.001).

In total, 521 differentially expressed genes were identified for strain *ch* cultured at 37°C vs. 39°C for 1 h (Fig. 6A). The gene expression levels of *6-phosphofructokinase 1* (CXQ85_004264), *6-phosphofructokinase 2* (CXQ85_000664), *RAG1* (CXQ85_005028), and pyruvate kinase (CXQ85_003665), which are involved in pyruvate production via sugar fermentation and hydrolysis, were up-regulated. However, gene expression of *pyruvate dehydrogenase* (CXQ85_001057), a key enzyme of the pyruvate dehydrogenase complex, was down-regulated (Fig. 6B). The results of RT-qPCR analysis confirmed that dynamic changes to the expression levels of genes were associated with the response of strain *ch* to heat stress (Fig. 6C through E).

### Mechanisms of strains *cau*, *cd*, and *ch* in response to heat stress

**Fig 7:**
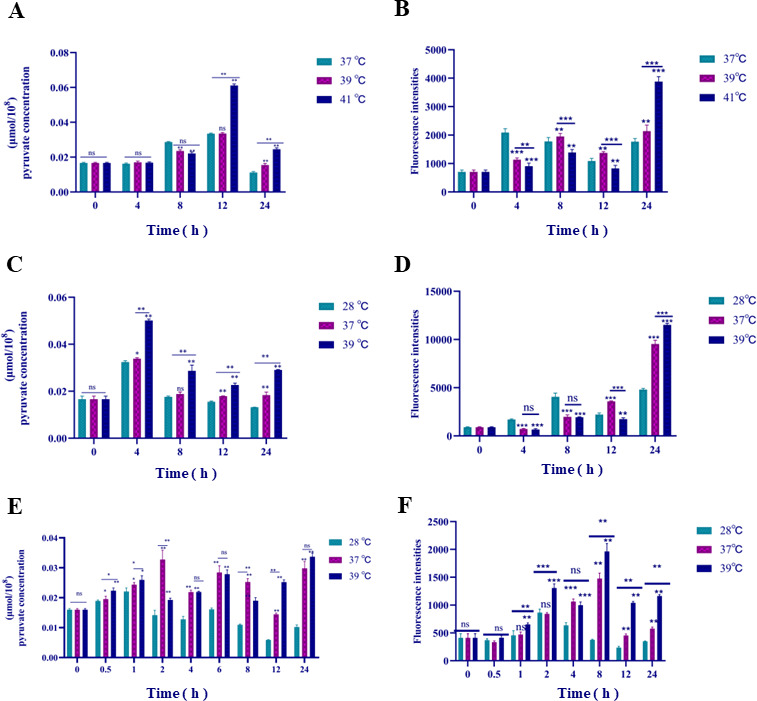
Changes to intracellular pyruvate and ROS levels in fungal cells in response to heat stress. Intracellular pyruvate (**A**) and ROS (**B**) levels of strain *cau* in response to heat stress. Intracellular pyruvate (**C**) and ROS (**D**) levels of strain *cd* in response to heat stress. Intracellular pyruvate (**E**) and ROS (**F**) levels of strain *ch* in response to heat stress. Data are the mean  ±  standard deviation of three independent experiments. (*P  <  0.05; **P  <  0.01; ***P  <  0.001; ns, not significant).

**Fig 8:**
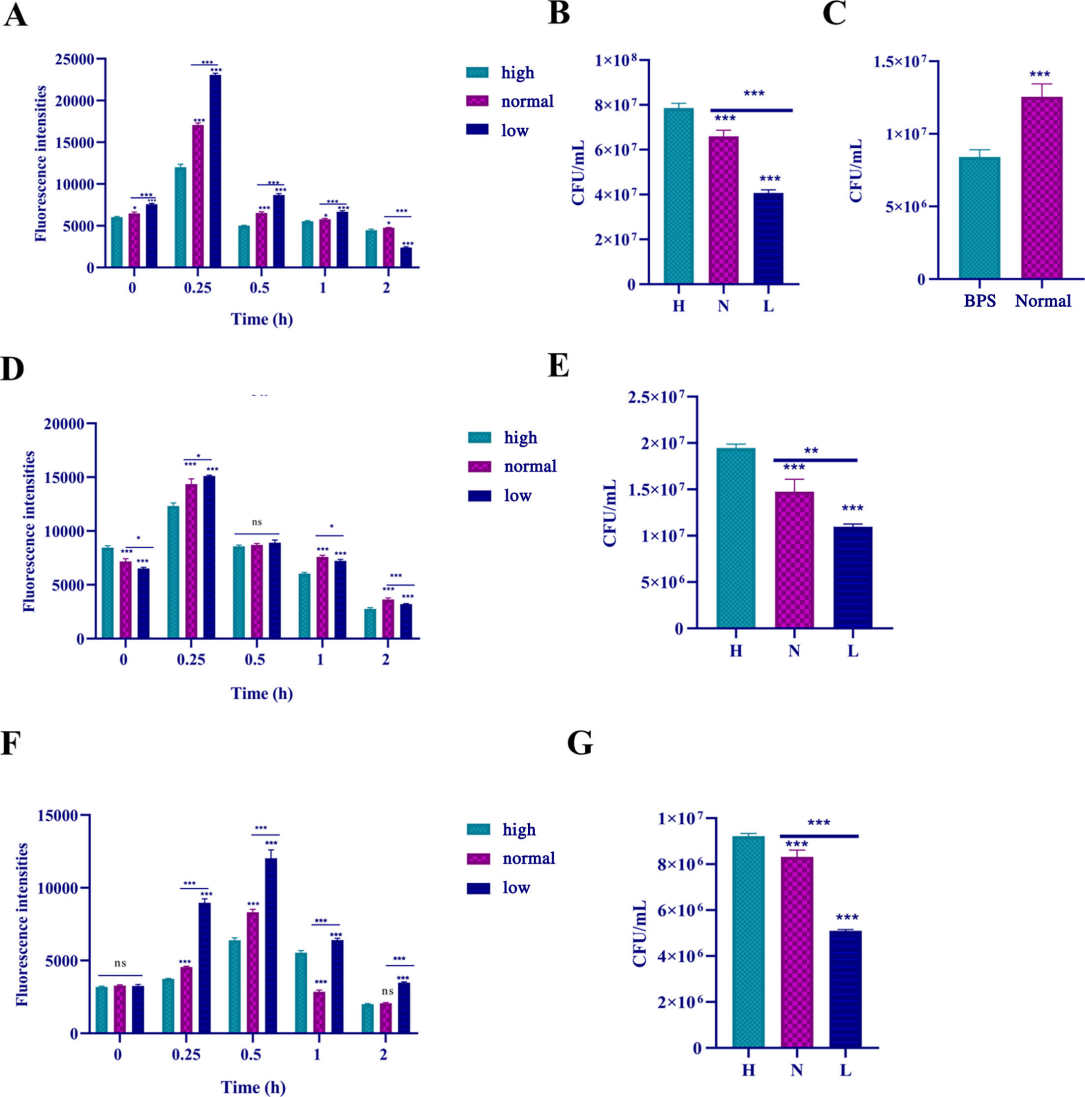
Changes to heat tolerance of fungal cells at different pyruvate concentrations. Pyruvate and pyruvate inhibitors were added to the culture medium to adjust pyruvate concentrations. Pyruvate was prepared at three concentrations: high (YPD liquid medium supplemented with 6 mM pyruvate), normal (normal YPD liquid medium), and low (YPD liquid medium supplemented with 4 µg/ml of the pyruvate kinase inhibitor PKM2-IN-1). (**A**) ROS levels of strain *cau* spores at different pyruvate concentrations in response to heat stress. (**B**) Population variation of strain *cau* spores with different pyruvate concentrations in response to heat stress. (**C**) Changes to CFUs of strain *cau* grown on iron-deficient medium in response to heat stress. (**D**) ROS levels of strain *cd* spores with different pyruvate concentrations in response to heat stress. (**E**) Population variation of strain *cd* spores with different pyruvate concentrations in response to heat stress. (**F**) ROS levels of strain *ch* spores with different pyruvate concentrations in response to heat stress. (**G**) Population variation of strain *ch* spores with different pyruvate concentrations in response to heat stress. Data are the mean  ±  standard deviation of three independent experiments. (*P  <  0.05; **P  <  0.01; ***P  <  0.001; ns, not significant).

To further verify the transcriptome data and RT-qPCR results, pyruvate concentrations of strains *cau*, *cd*, and *ch* in response to heat stress were measured. The results showed that the pyruvate contents and intracellular ROS levels of all three strains were increased with increasing temperatures (Fig. 7A through F), suggesting that the accumulation of pyruvate contributes to clearance of ROS. Subsequently, the ROS levels and survival rates of cells with various concentrations of pyruvate in response to heat stress were measured. The results showed that the strains treated with a pyruvate kinase inhibitor had the highest levels of intracellular ROS and the lowest survival rate, while the strains cultured with pyruvate had the lowest levels of intracellular ROS and the highest survival rate (Fig. 8A through F).

A previous investigation confirmed that the addition of iron to culture medium enhanced tolerance to oxidative stress and growth recovery of *Saccharomyces cerevisiae* (42). Similarly, enhanced iron uptake increased resistance of strain *cau* to heat stress, while iron-deficient medium significantly decreased proliferation (Fig. 8G). These results further confirmed that enhanced iron uptake improved resistance of strain *cau* to heat stress.

## DISCUSSION

**Fig 9:**
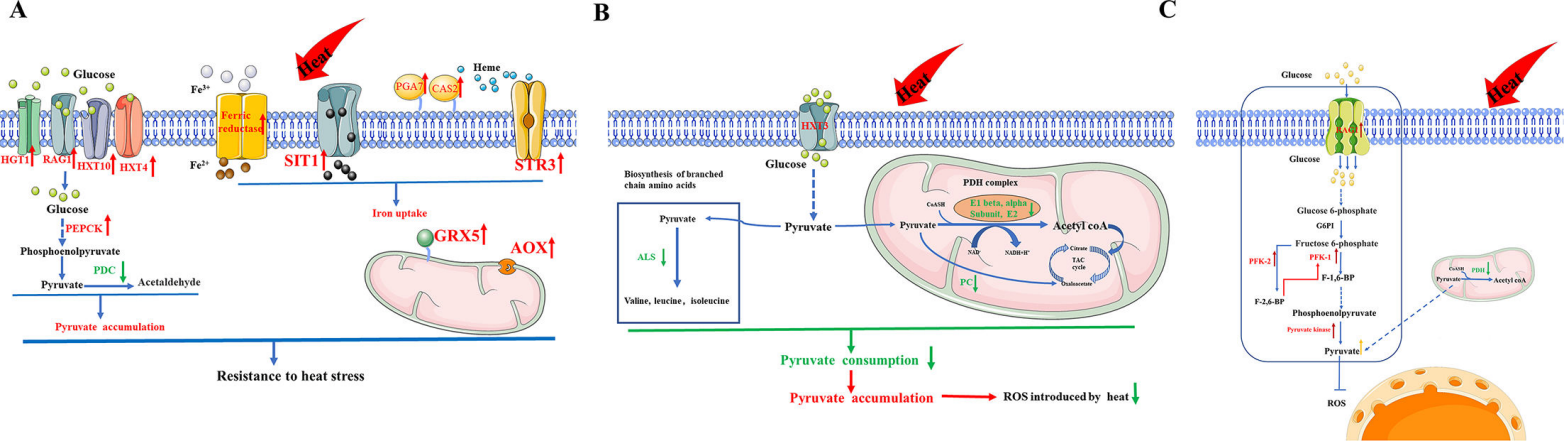
Schematics of proposed heat resistance mechanisms. (**A**) Heat resistance mechanism of strain *cau*. In strain *cau*, intracellular ROS levels were decreased by pyruvate accumulation and up-regulation of oxidase activity, and tolerance to heat stress was enhanced by up-regulation of iron uptake and utilization. (**B**) Heat resistance mechanism of strain *cd*. In strain *cd*, glucose uptake was accelerated and pyruvate consumption was decreased, thereby inducing intracellular pyruvate accumulation and reducing intracellular ROS, thus alleviating oxidative damage caused by heat stress. (**C**) Heat resistance mechanism of strain *ch*. Strain *ch* can resist heat stress by accelerating glucose uptake and up-regulation of multiple pyruvate-producing genes, thus inducing intracellular pyruvate accumulation.

Infection by drug-resistant fungi significantly increases the risk of mortality of severely ill hospitalized patients (43). Moreover, the relatively recent emergence of drug-resistant pathogenic fungi, including *C. auris* and *C. haemulonii* complex, has led to more severe nosocomial infections (44, 45). To reveal the mechanisms underlying pathogenicity, heat adaptation, and drug resistance, in this study, we first investigated the biological characteristics of different isolates, including drug resistance, virulence, and heat tolerance. Then, based on differences in heat tolerance, the protective mechanism of pyruvate accumulation was explored. The results revealed differences in the mechanism underlying resistance to heat stress (Fig. 9) between *C. auris* and *C. haemulonii* complex strains.

More than 4,000 cases of *C. auris* infection with high resistance to FLC, moderate resistance to AMB and CAS, and high sensitivity to MCF and ANI have been reported in 33 countries (46, 47). The resistance rates of *C. auris* to FLC, AMB, ANI, CAS, and MCF are reportedly 91%, 12%, 12.1%, 0.8%, and 1.1%, respectively (45). *C. haemulonii* complex exhibits resistance to multiple azole antifungal drugs (48). In the present study, the tested strains were commonly resistant to FLC (Table 1). Most strains of *C. haemulonii* complex in a prior study were sensitive to echinocandins, with very few exhibiting resistance (49). Two strains of *C. haemulonii* complex in our study were resistant to echinocandin drugs, indicating further evolution of multidrug resistance.

The virulence of *C. auris* and *C. haemulonii* complex was further determined through *in vitro* and *in vivo* experiments, which will contribute to future studies of the pathogenic mechanisms. Similar to a previous investigation (31, 50, 51), *C. auris* was more pathogenic than *C. haemulonii* complex in *G. mellonella* larvae, possibly due to the abilities of *C. auris* to avoid phagocytosis and form biofilms (52). In addition, consistent with the virulence of the three strains in *G. mellonella* larvae, *in vitro* analysis identified differences in adhesion, hydrophobicity, and biofilm formation among the four strains. Although all three strains secreted phospholipase, in contrast to previous studies, which reported that all *C. haemulonii* complex isolates secreted aspartic-type proteases (53).

Resistance to heat stress of the test strains was consistent with previous investigations, which showed that *C. auris* was more tolerant to high temperatures than members of the *C. haemulonii* complex (54). As a scavenger of ROS, pyruvate can effectively reduce protein carbonylation, stabilize mitochondrial membrane potential, and promote fungal growth (55). Previous investigations have suggested that heat stress can induce pyruvate accumulation in *A. fumigatus*, *C. albicans*, *Neurospora crassa*, and *S. cerevisiae* (55). Similar to previous investigations, our results confirmed that heat stress reduced intercellular ROS levels in the three strains caused by intracellular pyruvate accumulation. However, pyruvate accumulation and consumption differed among the tested strains at various temperatures (Fig. 7). Phosphoenolpyruvate carboxykinase converts oxaloacetic acid to carbon dioxide and phosphoenolpyruvate, which is then converted to pyruvate (56). Under non-oxidizing conditions, pyruvate decarboxylase catalyzes pyruvate to form acetaldehyde (57). In response to heat stress, the expression levels of genes associated with glucose uptake and pyruvate synthesis were up-regulated, while genes related to pyruvate consumption were down-regulated in strain *cau*. However, the mechanisms of pyruvate accumulation differed between strains *ch* and *cd* in response to heat stress. Pyruvate accumulation was achieved through up-regulation of genes related to the pyruvate synthesis pathway in strain *ch* (Fig. 6). Nevertheless, key genes of the pyruvate consumption pathway were down-regulated in strain *cd* (Fig. 5).

Iron is an essential nutrient for living organisms (58), whereas iron overload triggers ROS production (42, 59). An appropriate amount of iron can help yeast tolerate oxidative stress (58). In the present study, the expression levels of genes related to iron uptake and utilization, as well as the oxidase enzyme of stain *cau*, were significantly up-regulated in response to heat stress at all time points (Fig. 4). Iron plays an important role in the synthesis of pyruvate in cells by influencing the activity of key enzymes and its function in energy metabolism. Maintaining iron balance is crucial for ensuring the appropriate synthesis of pyruvate and the normal metabolic functions of cells. Therefore, accelerated iron uptake by strain *cau* in response to heat stress may increase activities of the tricarboxylic acid cycle as a protective mechanism to regulate pyruvate dynamics (58). In addition, iron supplementation of the *C. albicans* mutant strain *HSF1* had improved tolerance to multiple stressors (60). The RT-qPCR results confirmed that *HSF1* expression was up-regulated in strain *cau* in response to heat stress, suggesting that *HSF1* might regulate various genes related to iron uptake and utilization. However, further studies are needed to elucidate the underlying regulatory mechanism.

### Conclusions

In summary, the emergence of multidrug-resistant fungi is a serious challenge to healthcare globally. Although relatively few clinical isolates of *C. auris* and *C. haemulonii* complex were used in this study, this comprehensive comparative analysis revealed differences in the mechanisms underlying pathogenicity and protection against heat stress. *C. auris* strain resists heat stress by maintaining intracellular iron ion levels, accumulating pyruvate, and up-regulating the expression of the *GRX5* and *AOX* (aldehyde oxidase) genes. The *C. haemulonii* complex strains resist heat stress only by accumulating intracellular pyruvate. These data further revealed differences in the biological features of *C. auris* and *C. haemulonii* complex and provide useful references for clinical medication and prevention of *C. auris* and *C. haemulonii* complex infections.

## Data Availability

The authors confirm that the data supporting the findings of this study are available within the article. The data sets generated and/or analyzed during the current study are available in the Beijing Institute of Genomics Genome Sequence Archive (https://ngdc.cncb.ac.cn/gsub/) with the primary accession code no. CRA011199.

## SUPPLEMENTAL MATERIAL

Fig. S1 aem.02406-24-s0001.tif Growth activity curve of the strain at different temperatures over 120 h.

Table S1 aem.02406-24-s0002.docx Clinical information of patients with test strain and GenBank ID.

Table S2 aem.02406-24-s0003.docx Nucleotide sequences of primers used for RT-qPCR analysis.

ASM does not own the copyrights to Supplemental Material that may be linked to, or accessed through, an article. The authors have granted ASM a non-exclusive, world-wide license to publish the Supplemental Material files. Please contact the corresponding author directly for reuse.
