# Supplementary figures and images for: Comparative analyses of the biological characteristics, fluconazole resistance, and heat adaptation mechanisms of Candida auris and members of the Candida haemulonii complex
Source: Appl Environ Microbiol. 2025 Mar 26;91(4):e02406-24. doi: 10.1128/aem.02406-24 (PMC12016522; doi:10.1128/aem.02406-24)

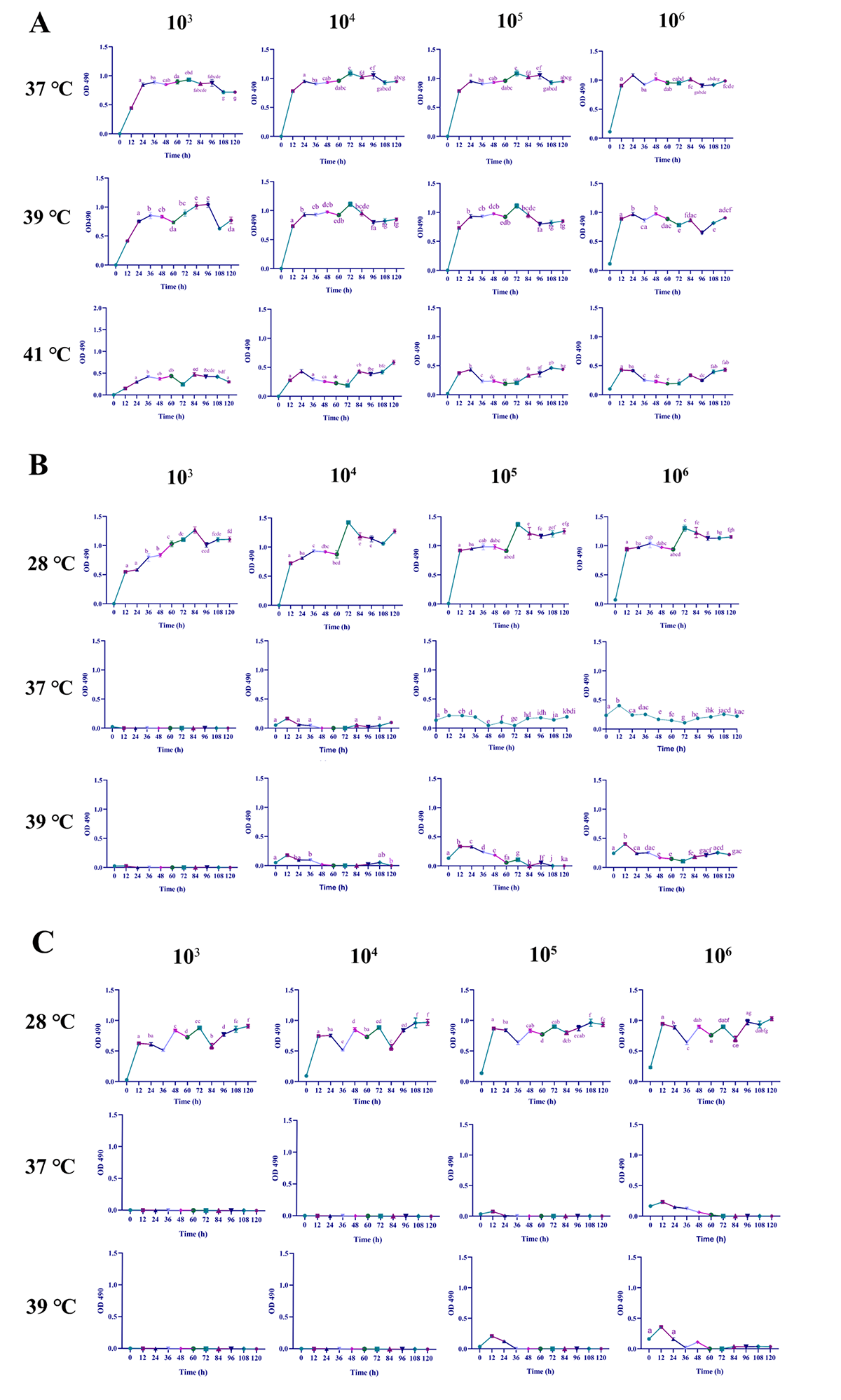

Supplement: Fig. S1 — Growth activity curve of the strain at different temperatures over 120 h. [file aem.02406-24-s0001.tif]
