# Supplementary material for: Comparative analyses of the biological characteristics, fluconazole resistance, and heat adaptation mechanisms of Candida auris and members of the Candida haemulonii complex
Source: Appl Environ Microbiol. 2025 Mar 26;91(4):e02406-24. doi: 10.1128/aem.02406-24 (PMC12016522; doi:10.1128/aem.02406-24)
Supplement: Table S1 — Clinical information of patients with test strain and GenBank ID. [file aem.02406-24-s0002.docx]

**Supplement Table 1.** Clinical information of patients with test strain and Genbank id

| **Strain** | **Source of isolate** | **Gender** | **Age (year)** | **Species** | **Genbank id** |
| --- | --- | --- | --- | --- | --- |
| *cau* | Blood | Man | 55 | *Candida auris* | PQ849753 |
| *cau03* | Blood | Man | 65 | *Candida auris* | PQ849754 |
| *cau13* | Blood | Woman | 57 | *Candida auris* | PQ849755 |
| *cd* | Blood | Man | 7 | *Candida duobushaemulonii* | PQ325703 |
| *cd1* | Skin | Man | 59 | *Candida duobushaemulonii* | PQ325704 |
| *cd2* | Blood | Man | 77 | *Candida duobushaemulonii* | PQ312707 |
| *cd3* | Blood | Woman | 65 | *Candida duobushaemulonii* | PQ312708 |
| *ch* | Blood | Man | 7 | *Candida haemulonii var* | PQ325705 |
