# Supplementary material for: Comparative analyses of the biological characteristics, fluconazole resistance, and heat adaptation mechanisms of Candida auris and members of the Candida haemulonii complex
Source: Appl Environ Microbiol. 2025 Mar 26;91(4):e02406-24. doi: 10.1128/aem.02406-24 (PMC12016522; doi:10.1128/aem.02406-24)
Supplement: Table S2 — Nucleotide sequences of primers used for RT-qPCR analysis. [file aem.02406-24-s0003.docx]

**Supplement Table 2.** Nucleotide sequences of primers used for RT-qPCR analysis.

| **Primer name** | **Sequence (5’-3’)** |
| --- | --- |
| *cau*-PAG7-F | TGTCCTTACTGGGACACTGG |
| *cau*-PAG7-R | CACAGAAGCAGGCATCATCC |
| *cau*-CAS2-F | CTGCCATTGAGTCTGCCATC |
| *cau*-CAS2-R | GGAAGAAGTGGTCTCCTGCT |
| *cau*-CAS1-F | TAGCTCTGCTACCGACTTGG |
| *cau*-CAS1-R | ACAGACACAATGCTGGAGGA |
| *cau*- str3-F | CCTCACGGTGTCCTTGTGTT |
| *cau*- str3-R | GCAGTATTGGCCATGGTTCC |
| *cau*-Sit1-F | TGGGTCCATTGCTTGGTTTG |
| *cau*-Sit1-R | GCACCGAAACCCATAACACA |
| *cau*-ARN1-F | AGGCCAGAAGAACTGATGCT |
| *cau*-ARN1-R | GGACTTGCAAGAACCTCGTC |
| *cau*-FeM-F | TAACGGAGGCAACAAAGCAG |
| *cau*-FeM-R | GGTGACGATTCCAAGCACTC |
| *cau*-Grx5-F | AGTGGCCTACGATTCCTCAG |
| *cau*-Grx5-R | TCCAACAAATCGGCCAACTC |
| *cau*- IND1 -F | GCCTACCGGTGAAACAGAAG |
| *cau*- IND1 -R | CTCGGTTCTCCACTGAGGTT |
| *cau*-PK-F | CAAGGCCATTGTCGTCTTGT |
| *cau*-PK-R | TCGACATCCTCTTGCCAGTT |
| *cau*-PDC-F | ATGGGTTCACCAGAGACCAG |
| *cau*-PDC-R | GAAGCAGGAACGGTCAAGTC |
| *cau*-RAG1-F | CGGTACCAAAGAGGCCTACA |
| *cau*-RAG1-R | CGTCAACACGAGACTTGGAC |
| *cau*-PC-F | TTGAGAGGTGCTAACGGTGT |
| *cau*-PC-R | CCAGCCTTCTTGACAGCATC |
| *cau*-AO-F | TACCATGGACAAGGCTGCAC |
| *cau*-AO-R | TTGTAGCCGGTTGCAATGTC |
| *cau*-AC-F | GAAGGAGATCACTGCTTTAGCC |
| *ch*-AC-F | TTATCAAGTGTGACGTCGATGTTC |
| *ch*-AC-R | GCAATACCTGGGAACAAAGTAGTA |
| *ch*-PDC-F | AGATCAGACAGGCCACCTTC |
| *ch*-PDC-R | AGCCAGAAACGTGAGATGGA |
| *cd*-PDC-F | ATCTTGGTCGATGCTTGTGC |
| *cd*-PDC-R | AGCTTCCTTCACATCTGGCT |
| *ch*-PDH-F | TTCGGACGATGGCATTGTTG |
| *ch*-PDH-R | GTGCATCGTGCACCAACTTA |
| *cd*-PDH-F | CGTTGGCCACTGTATTGAGG |
| *cd*-PDH-R | GACTTGACGATGGCAGGAAC |
| *ch*-PK-F | GTGTCATGTTGTCCGGTGAG |
| *ch*-PK-R | GTGCATCATGGAAACAGCCT |
| *cd*-PK-F | TAAGCCTGTCATCTGTGCCA |
| *cd*-PK-R | AATGGCGTTACCAACATCGG |
| *ch*-PFK1-F | CAGGCGCAGACTTCATCTTC |
| *ch*-PFK1-R | AAGCTCGTCGTCAATTGCTC |
| *ch*-PC-F | TTGAAGGCGATGTCCAACAC |
| *ch*-PC-R | AGTTGCTGAGCCTGGAAGAT |
| *cd*-PC-F | GAAGGGTCCAGATCCAGAGG |
| *cd*-PC-R | TGCTGAGCCTGGAAGATCAA |
| *ch*-RAG1-F | TTACTGGTCTTGCTGTCGGT |
| *ch*-RAG1-R | TGGCATCGTCGTACGTCTTA |
| *cd*-als-F | CTTGCCACCTTCTTGCCATT |
| *cd*-als-R | ACCGGTGGTGACAATAACCT |
| *cd*- HXT3-F | TCACCTATTCTGCCGAAGCA |
| *cd*- HXT3-R | CAATCTTGGCCAGGTGAAGG |
